# Aging Hematopoietic Stem Cells Decline in Function and Exhibit Epigenetic Dysregulation

**DOI:** 10.1371/journal.pbio.0050201

**Published:** 2007-07-24

**Authors:** Stuart M Chambers, Chad A Shaw, Catherine Gatza, C. Joseph Fisk, Lawrence A Donehower, Margaret A Goodell

**Affiliations:** 1 Program for Cell and Molecular Biology**,** Baylor College of Medicine, Houston, Texas, United States of America; 2 Stem Cells and Regenerative Medicine Center**,** Baylor College of Medicine, Houston, Texas, United States of America; 3 Department of Molecular & Human Genetics, Baylor College of Medicine, Houston, Texas, United States of America; 4 Department of Virology and Microbiology, Baylor College of Medicine, Houston, Texas, United States of America; 5 Department of Molecular and Cellular Biology, Baylor College of Medicine, Houston, Texas, United States of America; 6 Department of Pediatrics, Baylor College of Medicine, Houston, Texas, United States of America; The Salk Institute, United States of America

## Abstract

Age-related defects in stem cells can limit proper tissue maintenance and hence contribute to a shortened lifespan. Using highly purified hematopoietic stem cells from mice aged 2 to 21 mo, we demonstrate a deficit in function yet an increase in stem cell number with advancing age. Expression analysis of more than 14,000 genes identified 1,500 that were age-induced and 1,600 that were age-repressed. Genes associated with the stress response, inflammation, and protein aggregation dominated the up-regulated expression profile, while the down-regulated profile was marked by genes involved in the preservation of genomic integrity and chromatin remodeling. Many chromosomal regions showed coordinate loss of transcriptional regulation; an overall increase in transcriptional activity with age and inappropriate expression of genes normally regulated by epigenetic mechanisms was also observed. Hematopoietic stem cells from early-aging mice expressing a mutant *p53* allele reveal that aging of stem cells can be uncoupled from aging at an organismal level. These studies show that hematopoietic stem cells are not protected from aging. Instead, loss of epigenetic regulation at the chromatin level may drive both functional attenuation of cells, as well as other manifestations of aging, including the increased propensity for neoplastic transformation.

## Author Summary

Aging is marked by a decline in function of the entire organism. The effect of age on the regenerative capacity of adult stem cells, which should rejuvenate tissues throughout life, is poorly understood. Bone marrow stem cells, also known as hematopoietic stem cells (HSCs), continuously regenerate the cells that comprise the blood, including the immune system, which fails with age. Here, we show that older HSCs were less able to regenerate the blood system than young HSCs. Paradoxically, the HSC number increased concomitantly, leading to no major difference in overall blood production, even though the immune system did exhibit some defects. To determine why these changes occurred, we looked at global patterns of gene expression in young versus old HSC. Stem cells exhibited an elevated inflammatory response and a decline in factors, called chromatin regulators, that orchestrate DNA accessibility and gene expression. Additional evidence supports the idea that loss of overall gene regulation (epigenetic regulation) is a major event during aging. Whereas much of aging research is concentrated on accumulation of mutations in DNA rather than on global regulatory mechanisms, we speculate that these epigenetic changes could drive many of the manifestations of age. This view also may explain the increased incidence of cancer with age.

## Abstract 3

In highly purified hematopoietic stem cells from mice aged 2 to 21 months, gene expression analysis indicates a deficit in function yet an increase in stem cell number with advancing age.

## Introduction

Somatic stem cells replenish many tissues throughout life. In general, they have slow turnover and reside in specialized niches, protected from the environment, so that only a few are activated at a time. Thus, stem cells are a defense against aging, replacing cells lost through attrition. If the rejuvenating effect of stem cells were perfect, senescing cells would be replaced indefinitely; but even in highly regenerative tissues such as the skin, the gut, and the hematopoietic system, age-related decline in function is well established [1]. Still unclear are the effects of aging on the stem cells themselves, which could contribute to inferior tissue repair.

Hematopoietic stem cells (HSCs) continuously replenish the blood and immune system throughout life. Data from mice support an age-related decline in stem cell function [1], suggesting that older HSCs are inadequate to cope with the demands of blood production. When limited numbers of aged hematopoietic progenitors are transplanted into young recipients under competitive conditions, they show an overall reduction in long-term repopulating potential [2]; in particular, lymphopoiesis is deficient, whereas myelopoiesis is enhanced [3,4]. Paradoxically, however, the total number of primitive progenitors has been reported to increase with age in the C57Bl/6 mice [2,5]. A recent study of aged hematopoietic stem and progenitor cells suggested that increased expression of particular proto-oncogenes may underlie some of these observed changes [4].

Although the previous studies varied widely, the findings provide compelling evidence for major age-related alterations in HSC function. To gain insight into the molecular mechanisms that underlie these deficits, we examined gene expression in HSC as a function of age on a genome-wide scale in normal and an early-aging *p53* mutant strain. These data provide a comprehensive molecular portrait of aging in HSC, and show that stem cell aging mirrors the aging of other tissues, marked by a dramatic inflammatory response, stress responses, and substantial alterations in the regulation of chromatin structure.

## Results

### Phenotypically Defined HSCs Increase in Number with Age and Possess a Functional Defect

**Figure 1:**
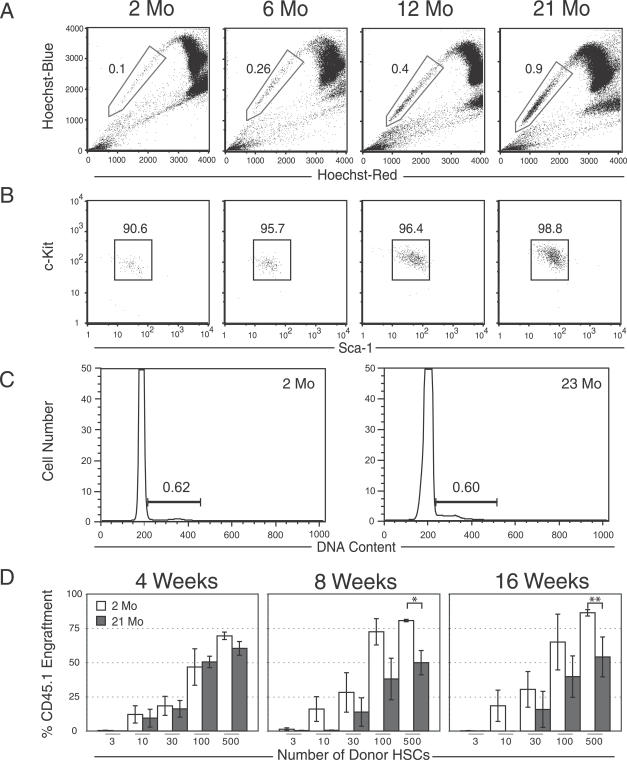
Aging HSC Phenotypes and Functional Alterations (A) Hoechst dye efflux by HSCs results in a SP (boxed) when viewed at two emission wavelengths. Comparison of the proportions of Sca-1–enriched SP cells from C57Bl/6 mice at 2 and 21 mo of age shows an approximate 9-fold increase with age. (B) Expression of the two canonical stem cell markers, c-Kit and Sca-1, does not change significantly between 2 and 21 mo of age within the lineage-negative (Lin^−^) SP population, indicating the SP cells remain remarkably phenotypically pure and homogeneous. (C) Cell cycle analysis by propidium iodide staining of 2- and 23-mo-old HSCs purified on the basis of SParKLS. (D) Limiting dilution functional assay of HSCs. In competitive repopulation experiments, there was little difference in HSC activity 4 wk after transplantation in young versus old HSCs. However, at 8 and 16 wk post-transplantation, 21-mo-old HSCs showed a reduced contribution compared to 2-mo-old control HSCs, depending on the donor cell dose (a single asterisk [*] indicates *p* ≤ 0.03; double asterisks [**] indicate *p* ≤ 0.09). Error bars represent one standard error.

The number of whole bone marrow (WBM) progenitors defined by cell surface markers in C57Bl/6 mice increases with age relative to total WBM cellularity [2,5]. To assess whether this property extends to HSCs purified using the side population (SP) cells defined by their ability to efflux Hoechst 33342 dye [6], we examined the SP cells in C57Bl/6 mice ranging from 2 to 21 mo of age. The results demonstrate a 9-fold increase in the number of SP cells with age (Figure 1A), with the most primitive SP^low^ cells [7] showing the greatest increase. These SP cells also exhibit the surface phenotype of HSC regardless of age (c-kit^pos^, lineage^neg^, and Sca-1^pos^ [SParKLS]), consistent with their high degree of purity and homogeneity (Figures 1B and S1). Aged HSCs were uniformly CD48^neg^, which is one of the markers recently described to mark differentiated hematopoietic cells from both young and aged mice [8,9] (unpublished data). Thus, murine HSCs defined by multiple phenotypes increase 9-fold in WBM over approximately 2 y. The increase in cell number was not a result of greater proportion of S-phase HSCs, as determined by propidium iodide staining (Figure 1C), suggesting an alternative mechanism for the increase in HSC number.

Limiting dilution bone marrow transplantation can measure the ability of HSCs to reconstitute recipients under competitive conditions and the functional purity of HSC [10]. HSCs (SParKLS) were therefore purified from either 2- or 21-mo-old mice and transplanted into lethally irradiated recipients, along with competitor bone marrow. Progeny from donor HSC were distinguished from competitor and recipient cells using the CD45 allelic system [11]. The proportion of peripheral blood progeny derived from purified young and old HSC was monitored at 4, 8, and 16 wk post-transplantation. Four weeks after transplantation, there was little difference in the contribution of young versus old HSCs (Figure 1D), but at 8 and 16 wk post-transplant, the contribution from the old HSCs was significantly reduced, but still multilineage (Figure S2). This finding argues that HSCs acquire a defect in long-term, but not short-term, repopulating potential with increasing age. This deficit represents roughly a 3-fold loss in functional activity per purified stem cell. With a 5- to 10-fold numerical increase in HSC, this indicates that the total stem cell activity remains fairly constant with age, which is consistent with other reports [2,12].

### Gene Expression Changes in Aging HSCs by Microarray Analysis

**Figure 2:**
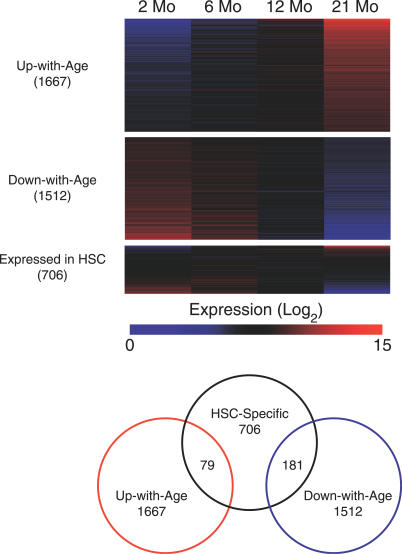
Gene Expression in HSC throughout Aging The heat maps show expression levels for four different gene lists, with the degree of overlap among the lists indicated on the right. Color intensity indicates level of expression, where blue signifies low expression and red signifies high expression. Each column delineates the mean expression at 2, 6, 12, and 21 mo, and each row represents a given gene within each gene list. “Expressed in HSCs” refers to genes derived from a comparison of HSCs versus WBM.

To identify transcriptional changes in aged HSCs that correlate with the observed functional deficit, we examined the expression of more than 14,000 genes, using Affymetrix MOE430a microarrays and HSCs purified from 2-, 6-, 12-, and 21-mo-old mice. A quadratic trend line (parabola) was fit for each gene over the 19-mo test period, which showed that the genes generally either increased or decreased in expression in a time-dependent fashion. We used a linear contrast model based on the entire observation course to determine which genes had the largest changes in expression over time. This revealed 1,600 genes that were up-regulated at 21 mo (“Up-with-Age” group), and 1,500 that were down-regulated (“Down-with-Age” group), which is summarized as a heat map in Figure 2. A small hand-picked list is shown in Table 1; the entire list of differentially expressed genes is supplemented in Tables S1 and S2, and a searchable database of all genes on the array can be found at http://rd.plos.org/pbio.0050201. Expression changes of a subset of these genes were validated by real-time quantitative PCR in duplicate on independently purified HSC (Figure S3). We also compared transcriptional profiles for WBM versus HSCs to identify HSC-specific transcripts; surprisingly, only a modest overlap of genes was found with those that were up-regulated or down-regulated with age, suggesting that the HSC-specific transcriptional programs remain relatively stable as the organism ages (Figure 2). A remarkable overlap was found between genes up- and down-regulated with age in this study and a previous study of HSC aging, with the top ten genes being identical [4].

**Table 1:**
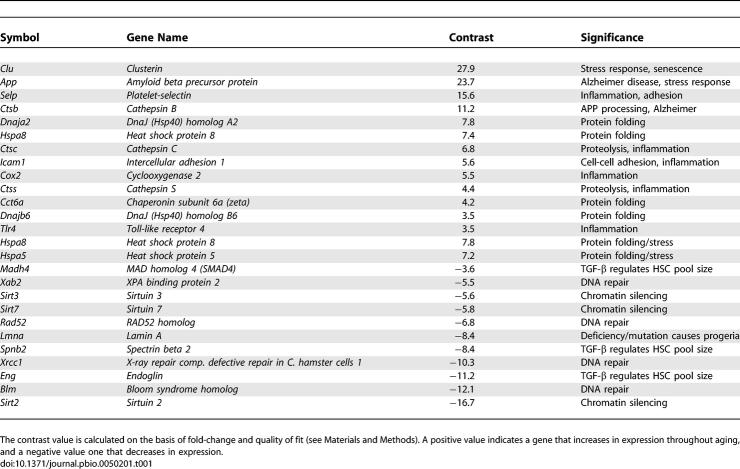
Selected Genes Differentially Expressed during HSC Aging

### Gene Ontology Categories Enriched for Age-Induced or Age-Repressed Genes

**Figure 3:**
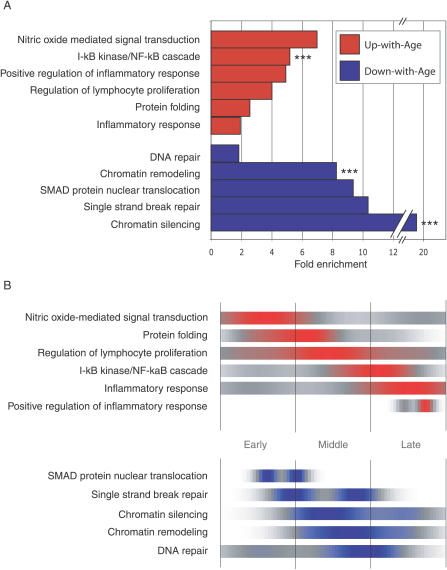
Gene Ontology Analysis (A) Fold enrichment over chance for selected GO categories of the Up-with-Age (red) and Down-with-Age (blue) gene lists. Bars without asterisks, *p*-value ≤ 0.05. Triple asterisks (***) indicate *p* ≤ 0.005. The number of genes found within each gene list and found on the entire array are shown for each GO category. (B) GO-timer *T* ^1/2-max^ for selected GO categories as a function of density over time. Areas of color correspond to the time at which a GO category is undergoing the most rapid up-regulation (red) or down-regulation (blue). It is important to note that after a given GO category *T* ^1/2-max^, the expression remains up-regulated (red) or down-regulated (blue).

We next sought to identify biological processes that were enriched in age-induced or age-repressed genes, compared to chance alone. For this purpose, we used Gene Ontology (GO; http://www.geneontology.org) to group genes on the basis of a particular biological process [13], and identified GO categories that were enriched with statistical significance by a method previously reported [14]. When applied to the Up-with-Age gene list, the analysis revealed a large number of enriched categories that have been linked to aging in general, such as NO-mediated signal transduction, the stress response (protein folding), and the inflammatory response, whereas categories enriched for Down-with-Age genes often included those involved in the preservation of genomic integrity, such as chromatin remodeling and DNA repair (Figure 3A) (the entire GO results can be found at http://rd.plos.org/pbio.0050201).

### NF-κB and P-Selectin Are Activated in Aged HSCs

**Figure 4:**
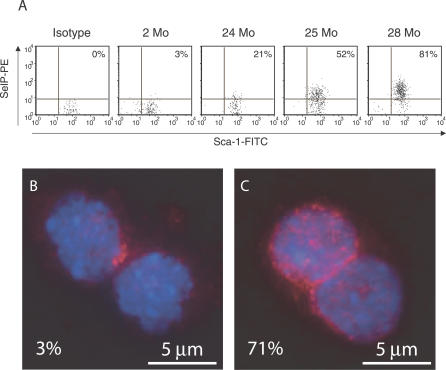
Up-Regulation of P-selectin Cell Surface Expression and NF-κB Localization in Aged HSCs. (A) An increasing percentage of HSCs express P-selectin (SelP) when examined by FACS, ranging from 3% (2-mo-old HSCs) to 81% (28-mo-old HSCs). FITC, fluorescein isothiocyanate; PE, phycoerythrin. (B and C) HSCs stained with anti-p65 NF-kB antibody (red) and DAPI (blue). Two-month-old HSCs contain approximately 3% nuclear-localized p65; however, at 22 mo, approximately 71% show nuclear-localized p65.

A link between aging and inflammation has been demonstrated in several vertebrate models and in humans [15], and we found evidence for the age-dependent regulation of several stress-related genes in HSCs. One of the most highly up-regulated of these genes expresses P-selectin, a cell surface adhesion molecule that serves as a marker for physiological stress states, including inflammation [16], aging [17], and cardiovascular disease [18]. P-selectin expression in HSCs, was of particular interest because it mediates the leukocyte–vascular endothelium interaction important for leukocyte extravasation [16] and therefore has implications for HSC migration. Flow cytometric analysis demonstrated increasing levels of P-selectin on the surface of HSCs isolated from 24- to 28-mo-old mice (21%–81%, Figure 4A), in contrast to scant levels (3%) on HSCs from young mice.

We hypothesized that the p65 isoform of NF-κB, which transcriptionally regulates P-selectin [19] would be activated in aged HSCs. To test this, we purified HSCs from 2- and 22-mo-old mice, and examined them for p65 localization by immunofluorescence. In contrast to only 3% of 2-mo-old HSCs, 71% of 22-mo-old HSCs showed enhanced nuclear localization of p65 protein (Figure 4B and 4C). These results implicate NF-κB activation as the mechanism of increased P-selectin expression in aged HSCs, most likely reflecting a time-dependent rise in inflammation.

### Timing of Gene Induction/Repression in Aging HSCs

The time course of data allowed us to examine the timing of changes in age-regulated gene expression. We determined when the trend line for each given gene achieved half its maximum change over the full time course (*T* ^1/2-max^), then grouped the genes by GO category and plotted the results for those categories that had a significant enrichment in the previous analysis (Figure 3A), creating a GO-timer. As shown in Figure 3B, genes that participate in NO-mediated signal transduction were the first to be up-regulated during HSC aging, followed closely by those contributing to the stress response and the regulation of lymphocyte proliferation. Inflammatory-response genes were not activated until late in the aging process, after up-regulation of NF-κB signaling, strengthening our hypothesis that inflammation exerts a strong influence on HSC aging through stimulation of the NF-κB pathway. Complete GO-timer results can be found at http://rd.plos.org/pbio.0050201.

### Centers-of-Regulated Expression Analysis

**Figure 5:**
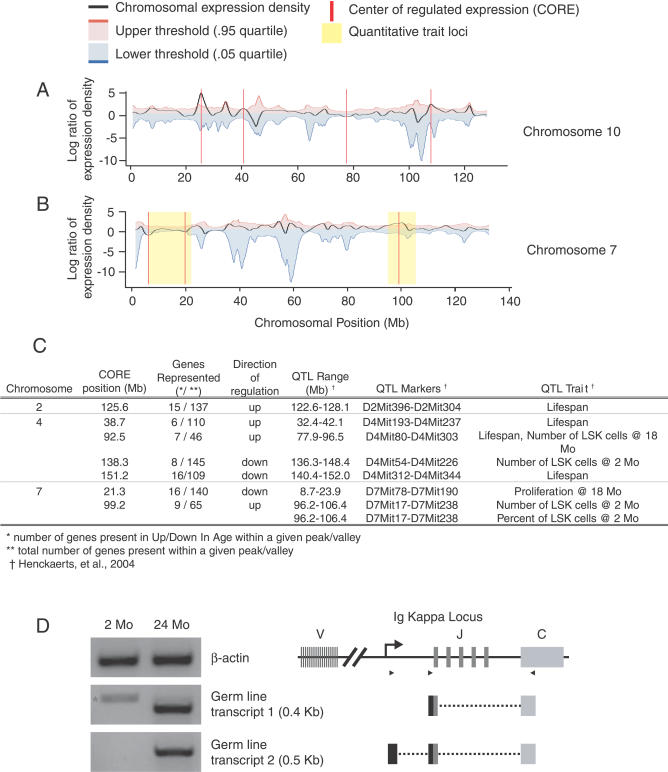
Density Plots of Coordinately Regulated Gene Expression for Chromosomes 4, 7, 10, and 13. (A) The black line represents the local density of coordinate regulation for all unique microarray probes. A positive value (peak) indicates a region where there are a greater number of up-regulated genes, whereas a negative value (valley) corresponds to a region of several down-regulated genes. The red vertical line indicates a CORE that extends beyond the threshold of significance (blue and red lines; *p* < 0.05) estimated by 1,000 randomized test sets. (B) Alignment of COREs on Chromosome 7 with previously published QTLs that represent chromosomal regions involved in age-related HSC function and organismal lifespan. Only those COREs that localize within established QTLs are shown. (C) Table showing the chromosomal alignment of COREs with known QTLs. QTLs are found on Chromosomes 2, 4, and 7 that contain COREs. (D) RT-PCR for *IgK* GL transcripts and diagram of transcript alignment with the GL *IgK* locus. The star (*) in the first lane, for GL transcript1, indicates a cDNA amplicon in young HSC that does not align with the *IgK* locus.

In *Saccharomyces cerevisiae,* the chromatin regulatory factor Sir2, a NAD-dependent histone deacetylase, suppresses recombination and silences transcription at multiple genomic loci [20]; its loss is associated genomic instability and aging. Since genes involved in chromatin remodeling and transcriptional silencing were excessively down-regulated in our GO enrichment analysis, we predicted global dysregulation of transcriptional activity. We reasoned that this would be evidenced by finding regions of chromosomes in which genes that were physically clustered together changed coordinately with age. To test this idea, genes were ordered by their chromosomal position, and age-induced and age-repressed genes were mapped using a density-based statistical approach. The result was a single curve across each chromosome, with peaks representing regions of coordinate up-regulation, and valleys regions of coordinate down-regulation (Figure 5A). Chromosomal loci with significant coordinate changes in gene expression were identified as centers of regulated expression (COREs; Figure 5A, red lines). Using this method, we found more than 100 such COREs among the 19 mouse autosomes (Table S3). Importantly, there were twice as many CORE peaks as there were valleys, indicating a predominance of a loss of transcriptional silencing throughout the genome.

Two recent linkage analyses in BXD mice indicated 22 chromosomal regions, or quantitative trait loci (QTLs), containing genes regulating hematopoietic progenitor number and organism lifespan [21,22]. We therefore asked whether any of the 17 mapped QTL loci might correspond to COREs. Eight of the QTLs mapped to seven COREs on Chromosomes 2, 4, and 7 (Figure 5C), consistent with the possibility that the QTL-associated genes on these chromosomes may play a critical role in regulating lifespan and HSC number. When compared to other non–HSC-related QTL datasets obtained from WebQTL [23], we observed a much greater overlap with the Henckaerts, et al. dataset [22] (*p*-value = 0.06).

### Immunoglobulin Kappa Expression Provides Evidence for Chromatin Dysregulation

With such dramatic changes in chromatin regulation, we expected to find inappropriate expression of specific genes in aged HSC. Indeed, with age, we observed a striking increase in expression of the immunoglobulin (Ig) heavy chain gene (*IgH;* ∼37-fold) and the immunoglobulin kappa *(IgK)* light chain gene (∼50-fold). Ig genes are not normally active in HSC, but become highly expressed in B cells. We thus decided to examine age-related transcription of the highly studied *IgK* locus in more detail. The possibility that B cells were contaminating the purified HSCs was excluded using antibodies against two markers of early and mature B cells, CD19 and Il-7r (Figure S4A). In addition, a PCR-based assay to detect Ig recombination [24] showed no evidence of V(D)J recombination (Figure S4B).

Transcripts from an unrecombined *IgK* locus, termed germline (GL) transcripts because they are removed during V(D)J recombination, have been sighted previously in a pre–pro-B cell line [25]; however, this occurs only after epigenetic modification, including DNA demethylation and subsequent histone modification [26]. Using reverse transcriptase PCR (RT-PCR) to detect GL *IgK* transcripts in poly-A RNA extracted from HSC from 2- and 24-mo-old mice, GL transcripts were readily detectable in HSC from old mice, but were absent in HSC from young mice (Figure 5D). Sequencing confirmed their GL structure, which included the entire first joining minigene (J1) and the constant region with a poly-A tail. These data are consistent with a loss of epigenetic regulation at the *IgK* locus with age in aged HSC. Such epigenetic changes may enable access by NF-κB and other factors that drive *IgK* gene expression, leading to inappropriate expression in aged HSC.

### Stem Cell Aging Is Uncoupled from Tissue and Organismal Aging in *p53* Mutant Mice

The tumor suppressor p53 is activated by stress and has been implicated in the balance between longevity and tumor formation [27]. Mutant mice expressing a truncated allele of *p53* (m allele) display an augmented p53 response, several early-aging phenotypes, and hematopoietic defects, including a blunted HSC proliferative response [28] and a lack of sufficient stem cell potential throughout life to maintain longevity [29]. In contrast, mice with half the amount of p53 *(p53^+/−^)* would be expected to live longer than wild-type (WT) mice, except that they develop lethal tumors. Consistent with the role of p53 in regulating cell proliferation, the *p53^+/−^* mice exhibit increased stem cell proliferation [29].

**Figure 6:**
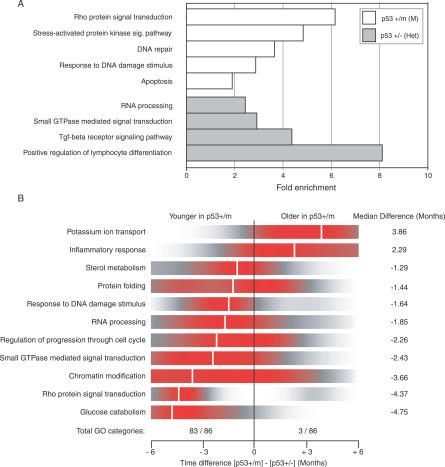
Gene Ontology for Pairwise Comparison and Age Differences between *p53^+/m^* and *p53^+/−^* HSC (A) A pairwise comparison between the two genotypes reveals differences in p53-regulated pathways, including DNA repair, Response to DNA damage, Apoptosis, and others. The number of genes found within each gene list and on the entire array are shown for each GO category. (B) Using the best-fitting WT HSC aging data (*R* ^2^ < 0.50), age was extrapolated on a per-gene basis for both the *p53^+/m^* and *p53^+/−^* expression data. Genes were clustered by GO categories, and categories with significant age differences between the two *p53* mutants were identified (Wilcoxon *t*-test; *p*-value < 0.05). Ninety-seven percent (84 of 87) of the GO categories were found to be younger (shifted left) in the *p53^+/m^* mice when compared to *p53^+/−^* mice. Shown is a density plot of the difference between the two genotypes on a per-gene basis for each GO category, where red indicates the highest density. Median differences in age for each GO category range from 1–5 mo and are demarcated by the white vertical line.

We examined HSC from *p53^+/m^* and *p53^+/−^* HSC to gain insight into the role of stem cells in organismal aging. HSC from 12-mo-old *p53^+/m^* and *p53^+/−^* mice were isolated and their gene expression profiles determined as above. These two strains studied constitute the available extremes of p53 activity; the *p53^+/−^* mouse has half the p53 dose of WT mice, and the *p53^+/m^* allele mice have *more* p53 activity than WT. A pairwise comparison between the *p53^+/m^* and the *p53^+/−^* HSC data (Tables S4 and S5) confirmed up-regulation of the GO categories DNA repair, Response to DNA damage stimulus, and Apoptosis in the *p53^+/m^* HSC (Figure 6A, Tables S6 and S7). Using the trend lines from the WT HSC time course, we calculated for each gene a predicted age (in months) based on the level of expression in both the *p53^+/m^* and *p53^+/−^* 12-mo-old mice. Interestingly, many of the genes from Table 1 exhibit a “younger” expression pattern in the *p53^+/m^* mice, including up-regulated genes such as *Icam-1* (−4.3 mo), *CatnB* (−3 mo), and *Dnaja2* (−2.8 mo), as well as down-regulated genes such as *Madh4* (−6.3 mo), *Hdac6* (−4.6 mo), *Dnmt3b* (−5 mo), and *Sirt7* (−6.4 mo). Genes were then grouped on the basis of GO, and the categories with a significant shift in predicted age (Wilcoxon *t*-test, *p*-value < 0.05) were identified. Overall, the *p53^+/m^* mice appeared younger by 1–5 mo compared to the *p53^+/−^* mice in the vast majority of significant GO categories (84/87) (Figure 6B and Table S8). Only three categories were “older” in *p53^+/m^* mice, the most striking being that of the inflammatory response, indicating that this can be uncoupled from the other manifestations of aging. Examples are P-selectin and Cox2, which are older in the *p53^+/m^* compared to the *p53^+/−^* HSC by +4.6 and +6 mo, respectively. The notion of relative youth of *p53^+/m^* stem cells is supported by the observation that HSC from *p53^+/m^* mice do not exhibit an age-related increase in number as is observed in their WT counterparts [30], and have attenuated activity after 5FU treatment [28], which can be explained by the lower HSC proliferative capacity. This is also consistent with our observation that HSC from the *p53^+/m^* mice form in vitro colonies at a similar frequency as WT HSC, but with reduced size, indicating a reduced proliferative capacity (Figure S5). These results suggest that although *p53^+/m^* mice appear older at a tissue and organismal level, the genetically imposed slower rate of proliferation uncouples the manifestations of aging in HSC, causing them to remain relatively young at a molecular level despite an inflammatory and aged tissue milieu.

## Discussion

Limitations in stem cell number or function have been proposed to restrict longevity. However, we show here that in WT mice, stem cells decline in function, when measured per HSC, with a concomitant increase in their number, resulting in a minimal net change in overall HSC activity, strongly suggesting that stem cells are not likely to be a factor limiting hematopoietic regeneration with age. However, their functional deficits do show that HSCs are impacted by the forces of aging in a manner similar to that of differentiated cells [31–34].

In our molecular analysis, we identified global age-related changes in gene expression in murine HSCs, with a view to identifying mechanisms that could be responsible for these age-associated declines in HSC function. Genes involved in inflammatory and stress responses dominated the group of up-regulated genes, whereas those participating in chromatin regulation and DNA repair were prominent among down-regulated genes.

Many of the observed expression changes are corroborated by a recent study of HSC aging [4]. Although the number of genes that go up or down with age was similar between our study and the one by Rossi et al. [4], the precise overlap between the genes is modest: only 343 genes were differentially expressed in both lists. We believe this is primarily due to a different purification strategy and array analysis that was used in our study. Remarkably, however, the top ten genes in both lists of genes that were Up-with-Age as well as those that were Down-with-Age were identical, and the magnitude of the changes was striking. For example, P-selectin was found highly up-regulated with age, for a mean fold-change of approximately 60 between the two studies. Likewise, both studies observed the up-regulation of specific protooncogenes, such as *Runx1,* that we believe could contribute to the increased incidence of myeloid leukemia with age.

In both studies, the inflammation markers P-selectin and clusterin (both regulated by NF-κB) and protein-folding genes such as *Hspa8* and *Dnajc3* were found up-regulated. Additionally, we found genes involved in DNA repair and chromatin maintenance *(Xab2, Rad52, Polb,* and *Lmna)* to be down-regulated in both studies, supporting the notion that HSC become epigenetically dysregulated.

The marked stress response exhibited by the HSC suggests that a proinflammatory microenvironment exists within the aging marrow. Studies of aging arteries, brain, and kidney have also observed up-regulation of inflammatory markers [33–35], but could not distinguish between immune cell infiltration and intrinsic inflammatory response of the tissue. The up-regulation of *TLR4 (Toll-like receptor 4)* (Table 1) is especially intriguing because one of its principal signaling targets is NF-κB, which regulates a variety of genes involved in the inflammatory response, including prostaglandin E2 synthase (Cox-2) and the adhesion molecules ICAM1 and P-selectin [36] (Table 1). This molecular profile resembles the elevated inflammatory state previously described in older mice (“inflamm-aging” [37]); however, our mice were raised in specific-pathogen–free conditions, and thus the inflamm-aging can be independent of antigenic load. Strikingly, Inflammatory Response was one of the few GO categories found “older” in the *p53^+/m^* mice compared to the *p53^+/−^* mice. This correlates with the early-aging phenotype observed at the organismal level in *p53^+/m^* mice, and suggests that inflammation is an intrinsic effect of age.

The up-regulated inflammatory genes in aging HSCs may have mechanistic implications for the decline of HSC function. The ability of HSCs to engraft in bone marrow is influenced by their homing properties; since cell adhesion plays a critical role in the engraftment of HSCs, aberrant up-regulation of genes encoding P-selectin and ICAM1 might affect the ability of older HSCs to home to the bone marrow, disrupting repopulation. Likewise, down-regulation of genes in the TGFβ signaling pathway such as *SMAD4, endoglin,* and *spectrin b2* (Table 1), may explain the increased number of HSCs, because the TGFβ pathway influences HSC pool size [38].

Equally striking was the increased expression of clusterin (clu) and amyloid beta precursor protein (App) in aged HSCs (Table 1), because these proteins aggregate within plaques in the brains of Alzheimer's patients [39]. Cathepsin B, involved in APP [40] processing, also increased in aged HSCs (Table 1). Clusterin, containing chaperone homology, along with several chaperonin subunits (beta, gamma, zeta, eta, and theta) increased with age, as did stress-response genes *Hsc70/Hspa8, HSP90/Hspca, Bip/Hspa5,* and several *Hsp40* homologs *(Dnaja1, Dnaja2, Dnajb6, Dnajb10,* and *Dnajc3)* (Tables 1 and S1). These changes, as well as the approximately 6-fold enrichment in NO-mediated signaling genes (Figure 3), may be in response to oxidative stress, a common feature of aged cells [41]. Some of these genes were previously noted to increase with age in murine hearts and skeletal muscle, and to decrease in animals on dietary restriction [31,32].

Although inflammatory and stress-response genes increased with age, genes that ensure transcriptional fidelity declined with age. For instance, *lamin A* declined with age and is linked to normal human aging [42] and Hutchinson-Gilford progeria [43] (Table 1). Likewise, *Bloom syndrome homolog (Blm)* declined, mutations in which cause genomic instability, hypermutability, and cancer predisposition [44]. Multiple DNA repair genes, including *Rad52, Xrcc1,* and *Xab2* were also down-regulated. Because DNA damage is thought to be a driving factor in aging [45], a blunted DNA damage response could retard DNA repair, increasing the risk of retaining mutations, leading to malignant transformation, one of the hallmarks of old age.

It was surprising that HSCs from 12-mo-old early-aging *p53^+/m^* mice appeared molecularly younger than age-matched WT and *p53^+/−^* HSC. This suggests that a genetically imposed lower rate of stem cell proliferation, as seen in the *p53^+/m^* HSC, can reduce the apparent age of the HSC, despite their residence in an environment that exhibits other outward manifestations of aging [28]. But this reduced proliferative capacity also results in poorer hematopoietic regeneration activity, when the stem cells are examined at the population level [28]. In other words, lower HSC proliferation results in a more youthful stem cell, but poorer tissue regeneration, and consequently an aged phenotype; this indicates that stem cell proliferation and tissue regeneration are finely balanced to maximize longevity, so that cell cycle disruption results in an uncoupling of tissue and organismal aging from the aging of the resident stem cell.

Finally, three lines of evidence in our work indicate broad changes in epigenetic regulation with age. Several GO categories (Figure 3A) and specific genes involved in transcriptional silencing via chromatin regulation are down-regulated with age, such as the SWI/SNF-related chromatin remodeling genes *(Smarca4* and *Smarcb1),* as well as three histone deacetylases (Hdac1, -5, and -6) and a DNA methyltransferase (Dnmt3b). Because these changes largely occur mid-way through life (Figure 3B), they could easily be envisioned to underlie inappropriate expression of additional genes. In addition, the CORE analysis revealed many chromosomal regions coordinately changing with age, and suggested an overall loss of transcriptional silencing. Finally, inappropriate transcription from the *IgK* locus, known to be driven by NF-κB activity following epigenetic modification enabling accessibility of the locus, was observed in old, but not young, HSCs (Figure 5).

Together, these data suggest an epigenetic view of aging that readily explains how so many diverse effects of age are evident at molecular, cellular, and organismal levels, and contrasts with the assumption that accumulation of lesions in genomic DNA or mitochondria accounts for the major effects of aging. Chromatin dysregulation could be a primary force in aging; epigenetic changes in otherwise normal cells could drive the loss of overall cellular functionality, as well as lay a fertile ground for secondary genetic events that lead irreversibly to oncogenic transformation. In this model, inappropriate expression of protooncogenes, or down-regulation of tumor suppressors, could result in a pre-transformed state, similar to myelodysplastic syndrome, a notion corroborated by another study of aging in murine HSC [4] in which *Runx1, Pml,* and other protooncogenes were up-regulated with age. Likewise, the increased transcriptional accessibility of some loci may enable interactions between otherwise distant chromatin domains, enhancing the likelihood of chromosomal translocations. Of particular note, the *IgK* locus that we show is transcriptionally active in aged HSC, is a well-established translocation partner with the *Myc* protooncogene in the generation of hematopoietic malignancies [46]. Moreover, *IgK* alleles have been shown to be differentially located within the nucleus depending on their state of activation [47], which could result in their juxtaposition to oncogenes, increasing the likelihood of translocation [48]. The role of epigenetic changes in cancer formation is increasingly recognized [49]; here, we suggest that chromatin dysregulation is a natural result of environmental insults with age, and a primary driver of secondary effects of age, including malignancy. A systems approach to the ways in which inflammation, stress response, and epigenetic regulation are linked may be essential to understanding aging and cancer.

## Materials and Methods

### Mice and HSC purification.

All mice were housed in a specific-pathogen–free barrier and fed autoclaved acidified water and mouse chow ad libitum. C57Bl/6 CD45.1 mice were allowed to age to 2, 6, 12, and 21–28 mo. HSCs were isolated as those cells that displayed the SP phenotype of Hoechst 33342 efflux [50] and were SParKLS. Hoechst and antibody staining was performed as previously described [11]. Sca-1 magnetic enrichment was performed with a Sca-1-biotin antibody (eBioscience, http://www.ebioscience.com/) and antibiotin microbeads (Miltenyi Biotec, http://www.miltenyibiotec.com/en/default.aspx) on an AutoMACS (Miltenyi). The Sca-1–enriched cells were suspended at a concentration of 10^8^ cells/ml and incubated on ice for 15 min with strepavidin-Alexa488 (Molecular Probes, http://probes.invitrogen.com), c-Kit-phycoerythrin (PE; eBioscience), and antibodies against the lineage markers: PE-Cy5 conjugated Mac-1, Gr-1, CD4, CD8, B220, and Ter119 (eBioscience). Flow cytometric analysis was performed on a triple-laser instrument (MoFlow; Cytomation, http://www.dako.com). For cell cycle analysis, HSCs were sorted into deionized water containing 0.1% sodium citrate and 50 μg/ml propidium iodide (PI) and analyzed with a FACScan flow cytometer (BD Biosciences, http://www.bdbiosciences.com).

### Limiting dilution analysis.

Forty CD45.2 mice were transplanted with 3, 10, 30, 100, or 500 CD45.1 HSCs from either 2- or 21-mo-old mice along with 1 × 10^5^ CD45.2 WBM competitor cells. Engraftment was measured by peripheral blood contribution 4, 8, and 16 wk post-transplantation.

### Immunofluorescence studies.

After sorting, the HSCs were resuspended in 50% FCS in HBSS at a concentration of 25,000 cells/ml, and 0.2 ml of cell suspension was spun onto glass slides with a cytocentrifuge (Wescor, http://www.wescor.com) at 800 rpm for 4 min. Fixed slides were blocked for 1 h with 30% goat serum in PBS, stained for 1 h with polyclonal anti-p65 antibody (1:100 in PBS; eBioscience), and stained with secondary antibody (1:250 and DAPI 1:1,000). Texas Red background immunofluorescence was established with controls containing no primary antibody at an exposure time longer than 5 s. Texas red immunofluorescence images were photographed for 0.8 s.

### RNA purification and hybridization to microarrays.

Total RNA was isolated from 2.5–5 × 10^4^ HSCs (2–5 mice pooled per microarray) with an RNAqeuous kit (Ambion, http://www.ambion.com), treated with DNAseI, and precipitated with phenol:chloroform. This RNA was linearly amplified in two rounds of T7-based in vitro transcription (MessageAmp kit; Ambion), and labeled in the last round with biotin-conjugated UTP and CTP (Enzo Biochem, http://www.enzo.com). Amplified biotinylated RNA (20 μg) was diluted in fragmentation buffer (5X; 200 mM Tris-acetate [pH 8.1], 500 mM KOAc, and 150 mM MgOAc in DEPC water) to a final volume of 40 μl, incubated at 94 °C for 25 min, and stored at −80 °C. A sample was run on a 4% nondenaturing agarose gel to confirm an RNA fragment length of approximately 50 base pairs. The labeled RNA was hybridized to MOE430A chips according to standard protocols (Affymetrix, http://www.affymetrix.com); the chips were then washed and counterstained with PE-conjugated streptavidin and a biotinylated anti-streptavidin antibody. The raw image and intensity files were generated with MAS 5.0 software (Affymetrix). All microarrays passed several quality controls as previously described [14]. Normalization and model-based expression measurements were performed with GC-RMA [51] (http://www.bioconductor.org).

### Linear regression and contrast analyses.

To identify genes whose expression varied significantly over time, we fit smooth curves to gene expression profiles by regression analysis. A quadratic polynomial was fit to the profile of each gene by use of LIMMA [52], enabling us to analyze progressive changes as well as peaks and valleys in expression over time. The Up-with-Age and Down-with-Age gene lists were identified by applying a linear contrast model and modified *t*-statistics from an empirical Bayes procedure [52] coupled with a linear step-up [53] multiple-testing correction (to estimate and minimize the false discovery rate to less than 5%) and a fold-change criterion of at least 2-fold for the 19-mo study period. In short, this contrast model is a composite score of reproducibility (*t*-statistic, *p*-value ≤ 0.05) and fold-change (>2-fold), which simply conveys a degree of difference (contrast) in gene expression over time.

### Gene Ontology analyses.

To investigate the biological significance of the gene lists described above, we used GO (http://www.geneontology.org). GO is a controlled vocabulary that describes gene biological roles and is arranged in a quasi-hierarchical structure from more general terms to the more specific. After mapping each gene in the two lists to the GO tree structure, we determined the number of genes at or below any given node in the GO hierarchy and the amount of statistically significant enrichment (Fisher exact *p-*value) for each GO node relative to chance observation, using a previously developed procedure [54]. To assess the emergence and disappearance of enriched GO categories, we defined the time of half-maximal expression change (*T* ^1/2-max^) for each gene in each category over the time interval. For genes whose maximal expression values were outside the 2- to 21-mo interval, the *T* ^1/2-max^ was determined as the intermediate expression value between the expression at 2 and 21 mo. For genes whose extreme expression values were within the interval, the *T* ^1/2-max^ was determined as the intermediate expression value between the expression at 2 mo and that extrema. Genes were grouped by GO category, yielding reliable estimates of time of induction and reduction for a given biological process. We conducted this analysis separately for the Up-with-Age and Down-with-Age gene lists.

### CORE analysis method.

To identify COREs, we obtained the genome coordinates of the Affymetrix MOE430A array from the MM5 build of the UCSC Genome Browser. To compare the locations of age-induced or age-repressed genes, we divided all genes into two disjoint classes based on the sign of the 21-mo versus 2-mo contrast. Redundant probe sets were removed by grouping all probe sets by Entrez Gene annotation. Because not all probe sets for a single Entrez Gene identifier have the same sign for the contrast score, the sign of the mean value was assigned to the Entrez Gene identifier. To compare the positions of these locations, we constructed a Gaussian kernel density estimate by chromosomal position for genes that increased with age and genes that decreased with age. We then calculated a ratio of these density estimates for the two groups. This ratio represents the density of genes that increase (peak) or decrease (valley) with age. A permutation test was performed to estimate the *p*-value where gene locations were randomly swapped along each chromosome, maintaining gene density but randomizing direction of regulation (up/down), Density estimate ratios were calculated based on 1,000 random permutations. This calculation enabled us to estimate a threshold of statistical significance such that peaks and valleys (high densities of age-induced and age-repressed genes) exceeding the 0.025 and 0.975 permutation-based quantiles were judged to be statistically significant at an estimated *p*-value of 0.05.

### Immunoglobulin assays.

Purified cells were sorted into lysis/PCR buffer, and PCR was performed as previously reported [24]. For GL RT-PCR, approximately 20,000 HSCs from either young or old mice were sorted into HBSS, and RNA was isolated by the RNAqueous kit (Ambion). RT-PCR was performed with an oligo-dT primer and SuperScript II (Invitrogen, http://www.invitrogen.com) followed by 50 cycles of PCR. RT-PCR fragments were purified, cloned into the Topo 2.1 vector (Invitrogen), and sequenced. IgH recombination primers are previously published [24]. *IgK* GL transcript primers include 5′-CTTCAGTGAGGAGGGTTTTTG-3′ (forward 1), 5′-ACTATGAAAATCAGCAGTTCTC-3′ (forward 2), and 5′-CGTTCATACTCGTCCTTGGTC-3′ (reverse).

### *p53* Gene Ontology analysis.

To assess the age-related expression differences between the *p53^+/m^* and *p53^+/−^* mice, genes with best-fitting trend lines (*R* ^2^ > 0.50) from the WT HSC aging time course were selected, and a predicted age (in months) was extrapolated for each gene based on the level of expression for both the *p53^+/m^* and *p53^+/−^* 12-mo-old mice. Genes were grouped on the basis of GO for both phenotypes and the categories with a significant shift in age (Wilcoxon *t*-test) between the *p53^+/m^* and *p53^+/−^* mice were identified by a *p*-value ≤ 0.05 and a median aged difference of greater than 1 mo. Mice used in these experiments have been back-crossed onto the C57Bl/6 background for four or more generations.

### Datasets

All data can be downloaded from our Web site http://rd.plos.org/pbio.0050201. In addition, all microarray data files have been deposited in the Gene Expression Omnibus (accession number GSE6503).

## Supporting Information

Figure S1 Fluorescence Activated Cell Sorter Analysis of SP Cell Lineage Expression Young (2 Mo) and Old (21 Mo) SP cells express very low levels of differentiated cell surface lineage markers (Gr-1, Mac-1, B220, Ter119, CD4, and CD8).
(492 KB PDF)

Figure S2 Fluorescence Activated Cell Sorter Analysis of Peripheral Blood Contribution of HSC from 21-Mo-Old Mice after Transplant HSC from old mice reconstitute all three lineages of the peripheral blood including myeloid (Gr-1 and Mac-1), B cell (B220), and T cell (CD4,8) at both 4 and 16 wks post-transplant.
(704 KB PDF)

Figure S3 Real-Time PCR of selected Up-Regulated HSC Aging Genes Error bars represent standard error from two separate experiments. mRNA was purified from HSC sorted independently from the HSC used in the microarray studies.
(441 KB PDF)

Figure S4 CD19 and IL-7r Are Not Expressed on HSC and PCR-Based IgH Recombination Assay (A) Young (red line) and old (green line) HSC do not express CD19 or IL7r compared to WBM (black line) on the basis of fluorescence activated cell sorting (FACS).
(B) The presence of DNA by a-actin (“A”), IgH GL locus (“Ig”), and recombined locus (“R”) have been examined using PCR in several populations including spleenocytes, B cells (B220^+^ Mac-1^−^), myeloid cells (Mac-1^+^ B220^−^), 2-mo-old HSC, 21-mo-old HSC, and 21-mo-old myeloid cells. No recombination was detected in any HSC.
(2.3 MB PDF)

Figure S5 Single HSC Methylcellulose Assays Single HSC from WT, *p53 ^+/−^,* and *p53 ^+/m^* 12-mo-old mice were sorted into 96-well plates containing methylcellulose (M3434; Stem Cell Technologies, http://www.stemcell.com) and allowed to form colonies for 14 d. *p53 ^+/m^* HSC were found to give rise to significantly smaller colonies (a single asterisk [*] indicates *p*-value ≤ 0.004) when ten colonies were pooled and the average number of cells per colony was determined for 60 colonies (*n* = 6) for each genotype. All three genotypes formed colonies at approximately the same frequency as shown in the table based on the percent of wells containing a colony (96-well plate).
(484 KB PDF)

Table S1 Up-with-Age in HSC Gene List (311 KB XLS)

Table S2 Down-with-Age in HSC Gene List (292 KB XLS)

Table S3 Table for COREs (245 KB XLS)

Table S4 Genes Up in *p53^+/m^* Compared to *p53^+/−^* HSC (125 KB XLS)

Table S5 Genes Up in *p53^+/−^* Compared to *p53^+/m^* HSC (105 KB XLS)

Table S6 Gene Ontology Enrichment Results for Up in *p53^+/m^* HSC (58 KB XLS)

Table S7 Gene Ontology Enrichment Results for Up in *p53^+/−^* HSC (77 KB XLS)

Table S8 Gene Ontology Table of Age Differences between *p53^+/−^* and *p53^+/m^* HSC (24 KB XLS)

### Accession Numbers

Entrez Gene (http://www.ncbi.nlm.nih.gov/sites/entrez?db=gene) ID accession numbers for the genes discussed in this paper are *App* (11820), *Blm* (12144), *CatnB* (12387), *Cct6a* (12466), *CD150* (6504), *CD19* (12478), *CD45* (19264), *CD48* (12506), *c-Kit* (16590), *Clu* (12759), *Cox2* (19225), *Ctsb* (13030), *Ctsc* (13032), *Ctss* (13040), *Dnaja1* (15502), *Dnaja2* (56445), *Dnaja2* (56445), *Dnajb10* (56812), *Dnajb6* (23950), *Dnajc3* (19107), *Dnmt3b* (13436), *Dnmt3b* (13436), *Eng* (13805), *Hdac1* (433759), *Hdac5* (15184), *Hdac6* (15185), *Hdac6* (15185), *Hspa5* (14828), *Hspa8* (15481), *Hspca* (15519), *Icam1* (15894), *IgH* (111507), *IgK* (243469), *Il-7r* (16197), *Lmna* (16905), *Madh4* (17128), *p53* (22059), *p65* (19697), *Pml* (18854), *Rad52* (19365), *Runx1* (12394), *Sca-1* (110454), *Selp* (25651), *Sirt2* (64383), *Sirt3* (64384), *Sirt7* (209011), *Smarca4* (20586), *Smarcb1* (20587), *Spnb2* (20742), *Tlr4* (21898), *Xab2* (67439), and *Xrcc1* (22594).

## Abbreviations

CORE: center of regulated expression
GL: germline
GO: Gene Ontology
HSC: hematopoietic stem cell
Ig: immunoglobulin
QTL: quantitative trait loci
RT-PCR: reverse transcriptase PCR
SP: side population
SParKLS: c-kit^pos^, lineage^neg^, and Sca-1^pos^
*T*^1/2-max^: time at half of maximal expression
WBM: whole bone marrow
WT: wild-type
