# Supplementary material for: Aging Hematopoietic Stem Cells Decline in Function and Exhibit Epigenetic Dysregulation
Source: PLoS Biol. 2007 Jul 24;5(8):e201. doi: 10.1371/journal.pbio.0050201 (PMC1925137; doi:10.1371/journal.pbio.0050201)
Supplement: Supplementary file 1 [file pbio.0050201.sg001.pdf]

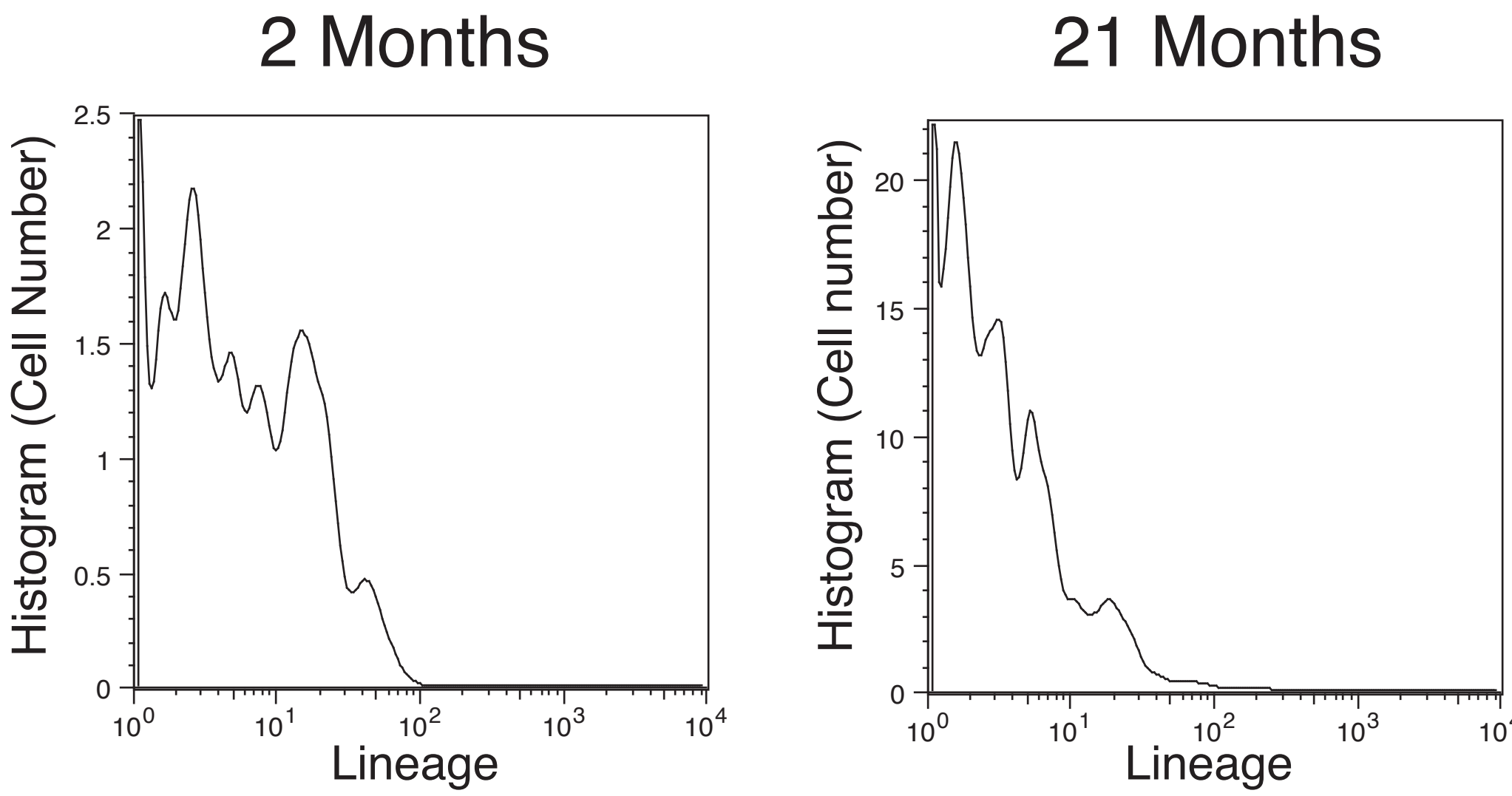

Supplemental Figure S1 - FACS analysis of SP cell lineage expression. Young (2 Mo) and Old (21 Mo) SP cells express very low levels of differentiated cell surface lineage markers (Gr-1, Mac-1, B220, Ter119, CD4, and CD8).
