# Supplementary material for: Aging Hematopoietic Stem Cells Decline in Function and Exhibit Epigenetic Dysregulation
Source: PLoS Biol. 2007 Jul 24;5(8):e201. doi: 10.1371/journal.pbio.0050201 (PMC1925137; doi:10.1371/journal.pbio.0050201)
Supplement: Supplementary file 2 [file pbio.0050201.sg002.pdf]

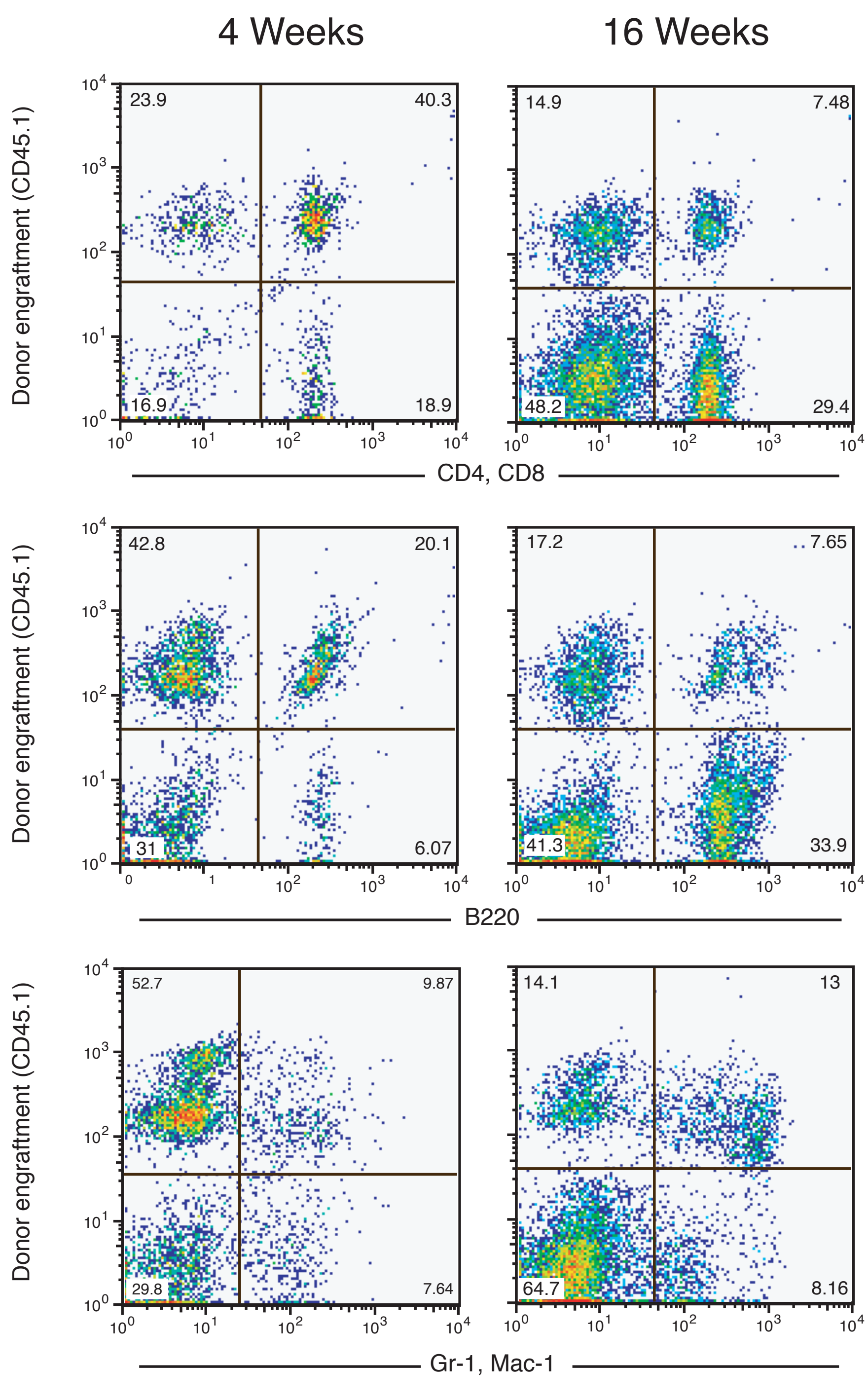

Supplemental Figure S2 - FACS analysis of peripheral blood contribution of HSC from 21-month-old mice after transplant. HSC from old mice reconstitute all three lineages of the peripheral blood including myeloid (Gr-1, Mac-1), B-cell (B220), and T-cell (CD4,8) at both 4 and 16 weeks post transplant.

Chambers et al.
