# Supplementary material for: Aging Hematopoietic Stem Cells Decline in Function and Exhibit Epigenetic Dysregulation
Source: PLoS Biol. 2007 Jul 24;5(8):e201. doi: 10.1371/journal.pbio.0050201 (PMC1925137; doi:10.1371/journal.pbio.0050201)
Supplement: Supplementary file 3 [file pbio.0050201.sg003.pdf]

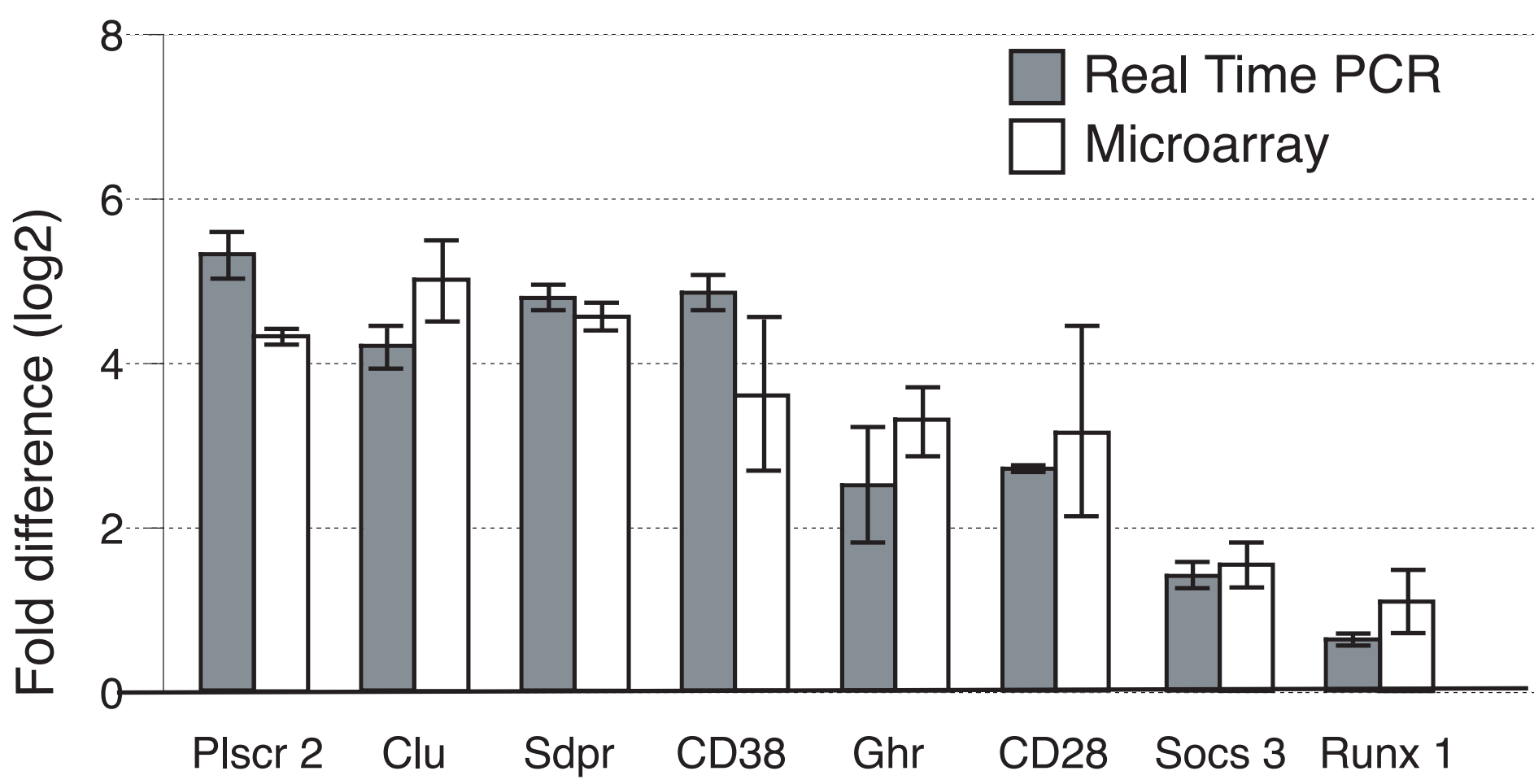

Supplemental Figure S3 - Real Time PCR of selected upregulated HSC aging genes. Error bars represent standard error from two separate experiments. mRNA was purified from HSC sorted independently from the HSC used in the microarray studies.
