# Supplementary material for: Aging Hematopoietic Stem Cells Decline in Function and Exhibit Epigenetic Dysregulation
Source: PLoS Biol. 2007 Jul 24;5(8):e201. doi: 10.1371/journal.pbio.0050201 (PMC1925137; doi:10.1371/journal.pbio.0050201)
Supplement: Supplementary file 4 [file pbio.0050201.sg004.pdf]

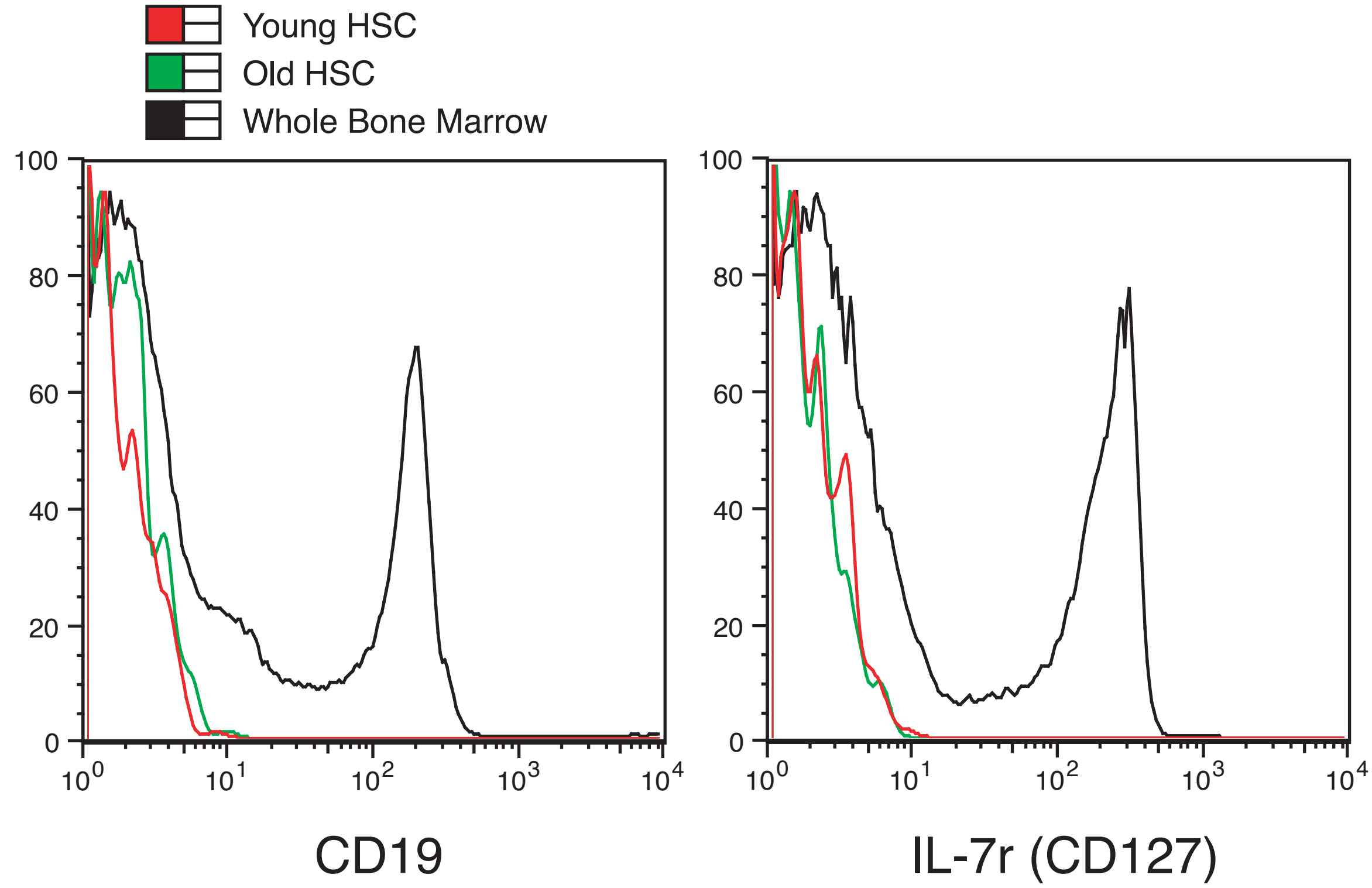

Supplemental Figure S4A - CD19 and IL-7r are not expressed on HSC. Young (red line) and Old (green line) HSC do not express CD19 or IL7r compared to whole bone marrow (black line) on the basis of FACS.

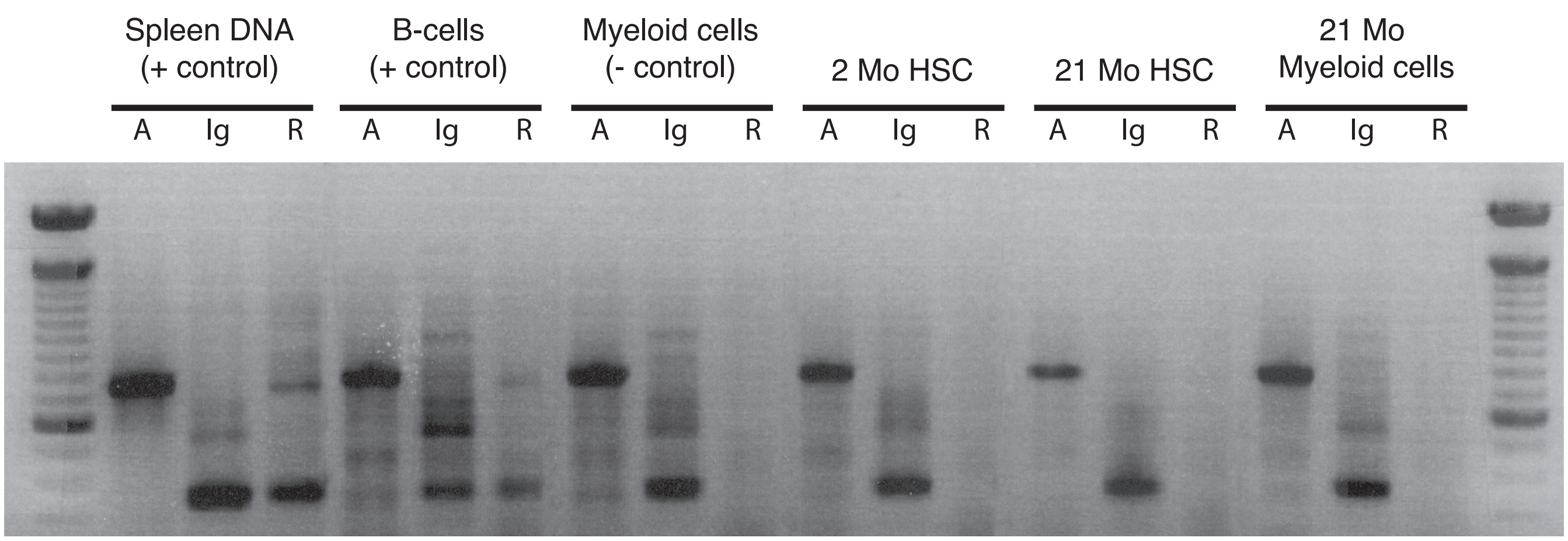

Supplemental Figure S4B - PCR-based Immunoglobulin (IgH) recombination assay - The presence of DNA by α-actin (A) , IgH germ-line locus (Ig) , and recombined locus (R) have been examined using PCR in several populations including spleenocytes, B-cells (B220+ Mac-1-), myeloid cells (Mac-1+ B220-), 2-month-old HSC, 21-month-old HSC, and 21-month-old myeloid cells. No recombination was detected in any HSC.
