# Supplementary material for: Aging Hematopoietic Stem Cells Decline in Function and Exhibit Epigenetic Dysregulation
Source: PLoS Biol. 2007 Jul 24;5(8):e201. doi: 10.1371/journal.pbio.0050201 (PMC1925137; doi:10.1371/journal.pbio.0050201)
Supplement: Supplementary file 5 [file pbio.0050201.sg005.pdf]

Chambers et al. Figure S5

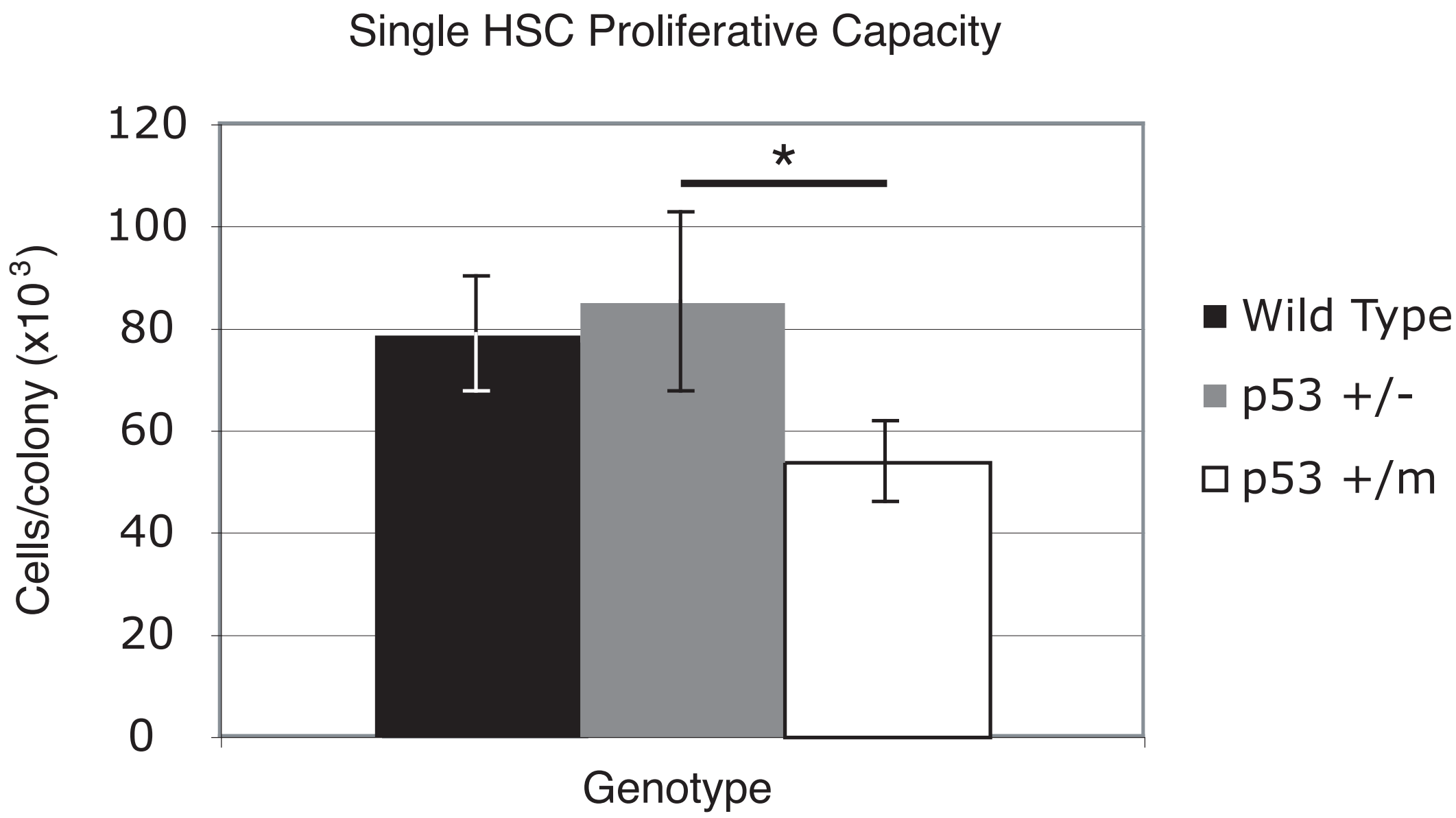

Percent of HSC forming colonies

	Plate 1	Plate 2
Wild Type	76	69
p53 +/-	84	84
p53 +/m	83	79

Supplemental Figure 5 - Single HSC from wild type, p53 +/-, and p53 +/m 12-month-old mice were sorted into 96-well plates containing methylcellulose (Stem Cell Technologies, M3434) and allowed to form colonies for 14 days. p53 +/m HSC were found to give rise to significantly smaller colonies (*p-value ≤ .004) when 10 Colonies were pooled and the average number of cells per colony was determined for 60 colonies (n=6) for each genotype. All three genotypes formed colonies at approximately the same frequency as shown in the table based on the percent of wells containing a colony (96-well plate).
